# Hemithyroidectomy for Thyroid Cancer: A Review

**DOI:** 10.3390/medicina56110586

**Published:** 2020-11-03

**Authors:** Noor Addasi, Abbey Fingeret, Whitney Goldner

**Affiliations:** 1 Department of Internal Medicine, Division of Diabetes, Endocrinology and Metabolism, University of Nebraska Medical Center, Omaha, NE 68198, USA; noor.addasi@unmc.edu; 2 Department of General Surgery, Division of Surgical Oncology, University of Nebraska Medical Center, Omaha, NE 68198, USA; abbey.fingeret@unmc.edu

**Keywords:** hemithyroidectomy, lobectomy, thyroid cancer, treatment, management

## Abstract

Thyroid cancer incidence is on the rise; however, fortunately, the death rate is stable. Most persons with well-differentiated thyroid cancer have a low risk of recurrence at the time of diagnosis and can expect a normal life expectancy. Over the last two decades, guidelines have recommended less aggressive therapy for low-risk cancer and a more personalized approach to treatment of thyroid cancer overall. The American Thyroid Association (ATA) and National Comprehensive Cancer Network (NCCN) thyroid cancer guidelines recommend hemithyroidectomy as an acceptable surgical treatment option for low-risk thyroid cancer. Given this change in treatment paradigms, an increasing number of people are undergoing hemithyroidectomy rather than total or near-total thyroidectomy as their primary surgical treatment of thyroid cancer. The postoperative follow-up of hemithyroidectomy patients differs from those who have undergone total or near-total thyroidectomy, and the long-term monitoring with imaging and biomarkers can also be different. This article reviews indications for hemithyroidectomy, as well as postoperative considerations and management recommendations for those who have undergone hemithyroidectomy.

## 1. Introduction

The incidence of differentiated thyroid cancer has increased significantly in the past few decades [1]. During 1994–2013, incidence-based mortality of thyroid cancer also increased 1.1% per year with a higher increase of 2.9% per year in patients with distant-stage papillary thyroid cancer [2]. However, the overall prognosis of thyroid cancer is still excellent with a 5 year relative survival rate of 98.3% [3]. Studies have shown that adjuvant therapy with radioactive iodine does not improve outcomes in low-risk thyroid cancers and often can contribute to long-term morbidity [4,5]. Similarly, less extensive surgical resection for selected cytologically indeterminate thyroid nodules and low-risk thyroid cancer patients has similar outcomes when compared to total thyroidectomy, with a lower risk of complication [4,5,6]. Current guidelines recommend hemithyroidectomy as an appropriate option for initial therapy for cytologically indeterminate thyroid nodules (Bethesda III and IV) and papillary thyroid carcinoma < 4 cm without high-risk features [4,6]. However, making the decision to recommend hemithyroidectomy rather than total thyroidectomy is often not as clear-cut as guidelines may suggest when counseling patients on the appropriate extent of surgery. There are additional factors to consider when recommending a hemithyroidectomy such as patient preference, potential for a completion thyroidectomy, need for postoperative adjuvant radioactive iodine (RAI) therapy, implications for long-term surveillance, and need for postoperative thyroid hormone replacement. Thyroid cancer is often diagnosed at a younger age and is associated with a normal life expectancy, especially in low-risk cancers; hence, it is important to not only focus on long-term morbidity, but also healthcare-related quality of life (HRQOL) when considering the most appropriate approach to the treatment of thyroid cancer.

The evaluation and treatment of thyroid cancer requires a coordinated team approach which consists of endocrinologists, surgeons, pathologists, radiologists, and occasionally medical oncologists. Physicians meet patients at different time points of their evaluation and treatment journey, and it is important that each member of the team is aware of the rationale for decision-making at each step of therapy. The purpose of this article is to review not only current indications for hemithyroidectomy, but also the pros and cons of hemithyroidectomy and indications for completion thyroidectomy; the long-term follow-up and management of those who have undergone hemithyroidectomy are also discussed.

## 2. Choosing the Initial Surgery

The extent of initial surgery should be individualized to each patient and should be based on a combination of factors: thyroid nodule imaging characteristics, fine-needle aspiration (FNA) cytology results, molecular testing if applicable, local symptoms, personal or family history of thyroid cancer, hereditary syndromes, history of radiation exposure, baseline thyroid hormone levels or presence of autoimmune thyroid disease, presence of contralateral thyroid nodules, and patient preference for short- and long-term management.

Hemithyroidectomy entails removal of the entire ipsilateral thyroid lobe and isthmus, with or without removal of central neck lymph nodes. Total thyroidectomy entails removal of the entire thyroid gland with or without removal of central neck lymph nodes. Whether hemithyroidectomy or total thyroidectomy is elected as the index operation, meticulous dissection to preserve the parathyroid glands and protect the recurrent laryngeal nerve is essential. Partial removal of the ipsilateral thyroid lobe is not recommended for the treatment of thyroid nodules or well-differentiated thyroid carcinoma. 

## 3. Preoperative Imaging Findings

### 3.1. Nodule Size

Size is an important consideration when determining appropriate thyroid surgery. Both the American Thyroid Association (ATA) and National Comprehensive Cancer Network (NCCN) thyroid cancer guidelines recommend consideration of hemithyroidectomy for nodules smaller than 4 cm without other worrisome features [4,6]. Active surveillance is an increasingly accepted option for small papillary thyroid carcinomas < 1–1.5 cm without evidence of suspicious features or lymph node (LN) metastasis [7]. Multinodular goiters causing compressive symptoms commonly require total thyroidectomy for symptomatic relief, if the enlargement affects the entire thyroid.

### 3.2. Suspicious Nodule Characteristics

Nodules that appear to have radiologic evidence of extrathyroidal extension (ETE) or invasion into adjacent structures suggest a higher risk of aggressive disease and local recurrence. In those cases, consideration of a total thyroidectomy as the initial surgery is important. If histopathology confirms micro or macroscopic ETE after hemithyroidectomy, this would be considered ATA intermediate- high risk of recurrence, and completion thyroidectomy with or without radioactive iodine ablation (RAI) may be indicated postoperatively. If ETE is suspected preoperatively, hemithyroidectomy may not be the best option, and recommending a total thyroidectomy could obviate the need for a second surgery.

### 3.3. Lymph Node Status

Thorough preoperative imaging of not only the thyroid gland, but also the central and lateral neck lymph node compartments is essential when evaluating thyroid nodules. The preferred imaging modality is ultrasound. Pathologic lymph nodes (LNs) in the central (level VI) or lateral lymph node compartments (levels II–V) should be biopsied, and thyroglobulin washings should be obtained from the needle rinse. If only the thyroid was imaged on initial evaluation, then lymph node mapping should also be performed preoperatively, especially for nodules with indeterminate or malignant cytology, and when surgery is planned [8]. Metastatic LNs indicate more advanced disease and the need for total thyroidectomy with appropriate lymph node dissection of the affected compartments.

## 4. Findings in the Contralateral Thyroid Lobe

If the contralateral lobe is normal, then initial hemithyroidectomy is appropriate if also meeting the above criteria. However, if the contralateral lobe contains nodules, then those nodules should also be evaluated preoperatively in order to make a comprehensive decision regarding the appropriate surgery. A thorough discussion with the patient about postoperative surveillance, especially if the histopathology shows malignancy, is important. Those with bilateral nodules will need more frequent postoperative surveillance imaging. This can increase patients’ anxiety, as well as add to long-term follow-up cost.

## 5. Bethesda Staging on FNA Results

The Bethesda System for Reporting Thyroid Cytopathology (TBSRTC) established a standardized reporting system for thyroid FNA specimens that was most recently updated in 2017. The six diagnostic categories are (1) nondiagnostic or unsatisfactory, (2) benign, (3) atypia of undetermined significance (AUS) or follicular lesion of undetermined significance (FLUS), (4) follicular neoplasm or suspicious for a follicular neoplasm, (5) suspicious for malignancy, and (6) malignant [9]. 

The ATA 2015 thyroid cancer guidelines favor a hemithyroidectomy for solitary thyroid nodules with Bethesda III or IV indeterminate cytology [4]. However, a total thyroidectomy is often preferred in patients with nodules that are cytologically suspicious for malignancy, positive for known mutations specific for carcinoma diagnosed via molecular testing, sonographically suspicious, or large (>4 cm) [4]. It can also be considered in patients with indeterminate nodules with bilateral nodular disease, those with significant medical comorbidities, or those who prefer total thyroidectomy to avoid a second operation assuming completion thyroidectomy is recommended if the indeterminate nodule is malignant [4]. The NCCN 2020 guidelines, on the other hand, offer a total thyroidectomy or hemithyroidectomy as treatment options for biopsy-proven papillary thyroid carcinoma (PTC) if the nodule size is < 4 cm, there are no cervical lymph node or distant metastases, and there is no ETE or prior radiation exposure [6].

Preoperative molecular testing results influencing the extent of surgery are very controversial. The mutation BRAFV600E is common in up to 60% of papillary thyroid carcinomas [10,11,12]. Some studies suggest that the presence of a BRAFV600E mutation predicts more aggressive behavior [12]. However, other studies suggest that it is present in both low- and high-risk tumors and should not influence extent of surgery, more than other associated clinical characteristics, especially showing that the BRAFV600E mutation status was not associated with increased risk of recurrence, response to therapy [13], or PTC-related mortality [14]. In contrast, TERT promotor mutations, alone or in combination with BRAFV600E mutations predict a high risk of recurrence or more aggressive disease course; thus, total thyroidectomy is recommended if these mutations are present [10,12,13,14]. However, most of the studies addressing the impact of these genetic mutations relied on retrospective analysis of previously treated patients [10,11,12,13,14], and more prospective studies will be needed to elucidate a clear role of these genetic markers in the risk stratification and subsequent management of patients with differentiated thyroid cancer.

## 6. Preoperative Thyroid Hormone Levels

One reason for patients’ preference for hemithyroidectomy is to avoid having to take postoperative thyroid hormone replacement therapy. Studies report variable rates of need for postoperative thyroid hormone replacement, ranging from 23.6%–73% [15,16,17,18]. Ha et al. reported a 50.3% euthyroidism rate without thyroid hormone replacement at 1 year postoperatively. The patients who were successfully maintained euthyroid off-therapy were mostly males, had lower thyroid-stimulating hormone (TSH) levels preoperatively, and had normal parenchymal histology of the thyroid gland [15]. Positive thyroid peroxidase (TPO) antibodies are associated with a higher chance of needing postoperative thyroid hormone replacement therapy. Lee et al. reported that 78% of patients with a preoperative TSH >2.5 and positive TPO antibodies needed thyroid hormone replacement compared to only 7% of the patients who had TSH levels < 2.5 and negative TPO antibodies [17]. Wilson et al. also reported remnant thyroid lobe volume adjusted for body surface area as a significant contributor to the postoperative need for thyroid hormone replacement therapy. The ratio of remnant volume to body surface area was higher in those who did not require thyroid hormone with 3.72 mL/m^2^ compared with 2.99 mL/m^2^ in patients who did require thyroid hormone supplementation [18]. 

Hence, when considering the need for postoperative thyroid hormone, it is important to look at higher predictors of needing postoperative thyroid hormone, such as preoperative TSH, echogenicity of the thyroid evaluating for heterogeneity, and presence of thyroid antibodies. If someone is already on thyroid hormone prior to surgery, they may be more inclined to have a total thyroidectomy. Additionally, the ideal TSH postoperatively will depend on the final pathology, as patients with malignancy will have a lower TSH goal than those with benign disease. The patient’s preoperative TSH level should be considered in concert with the likelihood of malignancy when counseling on the possibility of requiring thyroid hormone postoperatively.

## 7. Patient-Specific High-Risk Factors

Persons with a history of childhood neck radiation or familial thyroid cancer are at higher risk of cancer in the remaining thyroid tissue [4,19]. This should be factored into the choice of initial surgery with a total thyroidectomy being preferred. 

Another factor to consider is the overall health and operative risk profile. If the patient’s underlying health issues make their risk of a second surgery higher, then a total thyroidectomy may also be preferred as the initial surgery. Similarly, patients from remote or rural communities may prefer total thyroidectomy as their index operation if access to a high-volume thyroid surgeon requires distant travel.

## 8. Risk of Surgical Complications

Procedure-specific risks are also a consideration. Hemithyroidectomy is traditionally associated with lower rates of hypoparathyroidism [20]. Overall and procedure specific complication rates are lower if thyroid surgery is done by a high-volume surgeon [21]. In 2019, Nicholson et al. reported that operative outcomes for patients undergoing thyroid hemithyroidectomy, total thyroidectomy, and completion thyroidectomy were comparable including hematoma requiring reoperation, seroma formation, temporary vocal cord paralysis, and temporary hypoparathyroidism [22].

## 9. Patients’ Preference

Exploring patients’ preferences preoperatively and assessing their understanding of what each option entails in terms of clinical, biochemical, and radiological follow-up and the possible need for further surgical intervention is vital. It is also important for patients to understand that, even if the preoperative plan is to proceed with a hemithyroidectomy, there are intraoperative and postoperative findings that may require conversion to a total thyroidectomy. In a study of patients with low-risk thyroid cancer, the average patient favored hemithyroidectomy over total thyroidectomy as long as the chance of needing a second (i.e., completion) surgery after initial hemithyroidectomy remained below 30% and the risk of thyroid cancer was low, even if not zero. Patients also accepted a low risk of thyroid cancer recurrence [23].

## 10. Postoperative Follow-Up after Hemithyroidectomy

### 10.1. Determining the Need for Completion Thyroidectomy

This depends on multiple factors including the American Joint Committee on Cancer (AJCC) staging and the risk of recurrence per ATA criteria (see Table 1 and Table 2). If the clinician first meets the patient after they have already undergone hemithyroidectomy, then a review of preoperative imaging, FNA, and final histopathology results is necessary to determine the patient’s overall risk of recurrence. If adequate preoperative imaging demonstrating the contralateral thyroid lobe, as well as central and lateral neck compartments, was not done, this should be completed postoperatively. If there is concern for contralateral disease or abnormal lymph nodes, FNA of the suspicious areas should be performed with thyroglobulin (Tg) washings of lymph node (LN) biopsies. If there is evidence of structural disease, then completion thyroidectomy with appropriate lymph node dissection is indicated. If there is no evidence of structural disease, but the tumor has histopathological or other features with intermediate or high risk of recurrence according to the ATA criteria (see Table 2), then adjuvant therapy with RAI may be indicated. Thus, completion thyroidectomy should be strongly considered. Furthermore, if there is evidence of distant metastasis, then completion thyroidectomy and RAI are recommended [4,6].

Further management of a patient with a postoperative finding of a microscopic positive margin after hemithyroidectomy is controversial. Many studies have shown that the presence of microscopic positive margins does not increase the risk of recurrence in T1 and T2 low-risk tumors [26,27,28]. However, Lang et al. reported that the presence of a posterior microscopic positive margin did predict a higher risk of recurrence, thus raising the issue of whether this should prompt completion thyroidectomy with or without RAI [29]. The ATA does not designate positive margins as a risk factor for higher risk of recurrence [4]. However, the NCCN thyroid cancer guidelines recommend selective use of RAI, which would require completion thyroidectomy [6].

Multifocality in papillary thyroid cancer is common and has been estimated to involve 20% of cases [30]. Whether multifocality worsens prognosis and should dictate more aggressive treatment is still controversial. Earlier studies showed that patients with multifocal disease had a higher incidence of postoperative disease progression and lymph node metastasis [31,32]. This was more significant when the total tumor diameter was more than 1 cm [32]. However, more recently, Harries et al. reported that select patients with multifocal papillary thyroid cancer treated with hemithyroidectomy had similar rates of contralateral lobe papillary thyroid carcinoma, regional recurrence, and overall survival to those with unifocal disease treated with hemithyroidectomy [31]. These patients had T1/T2 N0M0 papillary thyroid cancer and had no evidence of contralateral lobe abnormalities [33]. It is unknown if this is also the case in larger tumors with multifocality. This is important to consider when deciding the extent of surgery. Patients found to have low risk of multifocal disease with normal contralateral lobes and no family history of thyroid cancer or personal history of head and neck radiation might not need a completion thyroidectomy. Prepublished data from our own institution, an academic tertiary care center in Omaha, Nebraska, showed a completion thyroidectomy rate of 24%, at a median of 25 days from index operation. Male sex and presence of bilateral nodules were associated with the need for completion thyroidectomy.

### 10.2. Follow-Up Labs and Scans

Follow-up of thyroid cancer usually consists of monitoring with ultrasound imaging of the neck and lab evaluation of thyroid function tests: mainly thyroid stimulating hormone (TSH) and tumor makers (thyroglobulin (Tg) and thyroglobulin antibody (TgAb)). If the contralateral lobe of the thyroid gland by ultrasound is normal and there are no abnormal LNs seen on imaging, then frequency of imaging can be reduced. TSH goals are generally low normal TSH (0.5–2.0 mIU/L) since those undergoing hemithyroidectomy by definition generally have a low risk of recurrence. Monitoring of Tg and TgAb should be considered but is not as predictive of recurrence after hemithyroidectomy as it is after total thyroidectomy. The monitoring of labs and scans has been termed the dynamic risk assessment.

### 10.3. Dynamic Risk Assessment

Because of the excellent survival rates in patients with thyroid cancer, especially those with low risk of recurrence, there was a need for a practical risk assessment tool for ongoing follow-up purposes. In 2010, Tuttle et al. suggested a dynamic risk stratification system for patients with thyroid cancer treated with total thyroidectomy followed by RAI ablation (Table 3) [25]. This tool was designed to be utilized at each follow up visit and categorized patient response to treatment into excellent, indeterminate, biochemically incomplete, and structurally incomplete [25]. However, residual normal thyroid tissue in patients treated with hemithyroidectomy can still produce Tg. Therefore, the same cutoffs cannot be applied to these patients. 

In 2014, Momesso et al. developed a modified risk stratification utilizing a Tg cutoff of 30 ng/dL for patients after hemithyroidectomy to define an excellent response to therapy (Table 3) [34], and this was validated in another study in 2016 [35]. A nonstimulated Tg cutoff of 30 ng/mL was chosen since a normal thyroid gland usually secretes 20–60 ng/mL and a lobe would be expected to secrete 50% of this [34]. The ATA 2015 thyroid cancer guidelines recommend considering checking serum Tg after hemithyroidectomy and then checking it every 12 to 24 months [3].

The exact Tg threshold to predict thyroid cancer recurrence, however, remains controversial. More recent studies showed a limited value of Tg as an independent predictor of thyroid cancer recurrence in patients with hemithyroidectomy [36,37]. In 2018, Park et al. reported a cohort of 208 patients with low-risk papillary thyroid cancer who underwent a hemithyroidectomy and were monitored for a median of 6.9 years. It was noted that serum Tg levels increased gradually by about 10% per year without evidence of recurrence, and there was no significant difference in Tg increase or Tg-to-TSH ratio between patients with or without recurrences. Similar findings were also reported by Ritter et al.; 167 patients with thyroid cancer treated with hemithyroidectomy were followed for a mean of 6.5 years. Some of the patients who developed recurrence had rising Tg, others had stable levels, and, in one patient, Tg antibody decreased despite metastatic lymph nodes [37]. They concluded that baseline Tg level post surgery and Tg trends did not predict disease recurrence [37]. One common feature in both cohorts is a very small group of patients with recurrent disease, which would be expected in a population suitable for hemithyroidectomy. However, it is unknown if it could have affected the ability to detect a statistical difference in Tg effect, and more studies are needed to answer this question. It would be helpful to have a biochemical marker to predict recurrence following hemithyroidectomy, to lessen the need for anatomic imaging; however, at this time, Tg does not unequivocally predict recurrence. The situation in which it may be more helpful is as a one-time check post hemithyroidectomy to rule out the presence of metastatic disease as signaled by an extremely elevated Tg. More studies will need to be done to assess the utility of Tg in this scenario.

Currently, periodic imaging of the neck with ultrasound is the preferred way to detect structural recurrence. Some clinicians check Tg and Tgab levels with yearly TSH, but the utility of this is debated.

## 11. Other Considerations

### 11.1. Noninvasive Follicular Thyroid Neoplasm with Papillary-Like Nuclear Features (NIFTP)

The indolent behavior of the encapsulated follicular variant of papillary thyroid carcinoma (EFVPTC) has been recognized for the past decade. In 2016, the Endocrine Pathology Society working group adopted a new nomenclature for the noninvasive EFVPTC, now known as noninvasive follicular thyroid neoplasm with papillary-like nuclear features or NIFTP [38]. NIFTP has a similar presentation to other thyroid neoplasms and is detected as a palpable nodule or incidentally found during imaging for other reasons [39]. FNA of these lesions is usually reported in categories III–V of the Bethesda system for reporting thyroid cytology [38]. However, NIFTP has a strict cytopathologic and histopathologic diagnostic criteria (Table 4). 

Completion thyroidectomy is not recommended for this type of tumor. NIFTP has an excellent prognosis, with extremely low risk of recurrence, even when treated conservatively with a hemithyroidectomy and no RAI therapy [38,39,40]. Xu et al. studied a cohort of large NIFTP, ranging from 4.0–8.0 cm, and showed similar outcomes to smaller lesions with zero risk of recurrence over at least 2 years of follow-up, including in the lesions treated with hemithyroidectomy and no RAI [40]. As a result, a conservative management is encouraged. However, the extent of long-term follow-up remains unknown. Most authors continue to recommend periodic imaging with unclear frequency [39].

### 11.2. Cost of Care of Thyroid Cancer Treated with Hemithyroidectomy vs. Total Thyroidectomy

The increased incidence and diagnosis of low-risk thyroid cancer combined with a stable mortality rate resulted in a large, growing pool of thyroid cancer survivors in need of surveillance. In 2015, Wang et al. estimated the cost of detecting a recurrence after total thyroidectomy for low-risk thyroid cancer at USD 147,819 [41]. Shrime et al. compared the 20 year cost-effectiveness of initial hemithyroidectomy vs. total thyroidectomy, including the cost of the initial operation, surveillance, and treatment of any recurrence, and found total thyroidectomy to be a more cost-effective option [42]. Similar results were reported by Leiker et al. in 2013 [43]. A comparison of low-risk thyroid cancer surveillance in the primary care vs. tertiary care setting favored primary care as a more cost-effective venue with similar outcomes [44]. The presence of residual normal tissue in patients with hemithyroidectomy and the controversy of tumor marker thresholds could possibly increase the need for imaging studies and the retention of these patients’ follow-up in tertiary centers. However, more studies are needed to evaluate this, especially as the current cost efficacy analyses do not account for the potential healthcare-related quality of life benefit or decrement.

### 11.3. Healthcare-Related Quality of Life (HRQOL)

Long-term surveillance is a key part of thyroid cancer management. An approach that addresses patient HRQOL in addition to survival benefits is essential. Studies have shown that even patients who have low-risk thyroid cancer with excellent prognosis had significant anxiety and worries related to their diagnosis [45,46]. This included worries about death, cancer recurrence, treatment side effects, and impact on quality of life and productivity. The anxiety that patients with thyroid cancer were reporting was comparable to those with cancer types with worse prognosis and outcomes [46]. Young age, female gender, lower education status, and being from a racial/ethnic minority increased risk of poorer QOL [45]. Since the ATA guidelines support hemithyroidectomy as an acceptable treatment option for low-risk thyroid cancer [4], there is movement toward a “less is more” approach to treatment of low-risk thyroid cancers. One study evaluated thyroid cancer treatment in elderly patients with low-risk papillary thyroid cancer and found that, despite changing recommendations, most patients still underwent total thyroidectomy and one-third of patients also received RAI. The patients with more aggressive therapy did not have a significant difference in disease specific survival compared to those with less aggressive management [47]. However, they had a higher risk of possible adverse effects from additional or more extensive surgery and RAI.

Chronic asthenia following total thyroidectomy has also been recognized as a possible contributor to worse QOL in thyroid cancer patients. Rosato et al. compared rates of asthenia following total thyroidectomy versus hemithyroidectomy and reported significant differences with 25% of the total thyroidectomy group reporting asthenia, compared with 0% of the hemithyroidectomy group [48]. Conversely, Bongers et al. reported no significant difference in long-term global health-related QOL for patients treated with hemithyroidectomy vs. total thyroidectomy for low-risk thyroid cancer, although patients with total thyroidectomy reported poorer cognitive and social functioning than those with hemithyroidectomy [46]. Their secondary analysis showed that worry about cancer recurrence was higher in the hemithyroidectomy group [46]. Patient-reported outcome measures for HRQOL may be subject to recall and selection bias as those with impaired HRQOL may be more apt to respond and more pronounced in their reported deficits. 

## 12. Conclusions

Hemithyroidectomy is a good treatment option for many low-risk thyroid cancers and is associated with less extensive surgery, lower rates of surgical complications, and a lower risk of postoperative asthenia in appropriately selected patients. When recommending hemithyroidectomy, it is important to ensure that adequate preoperative evaluation has been completed and that not only the short-term surgical plan is discussed, but also the long-term issues regarding thyroid hormone therapy, surveillance, need for additional surgery and follow-up, ability to predict recurrence, and QOL. Involving the patient in the discussion with the multidisciplinary team regarding appropriate treatment options is a great way to address all of these issues preoperatively.

## Figures and Tables

**Table 1:** American Joint Committee on Cancer (AJCC) Eighth Edition staging of differentiated thyroid cancer. T, tumor; N, node; M, metastasis.

Age at Diagnosis	T Staging	N Staging	M Staging	Stage
**< 55 years**	Any T	Any N	M0	I
	Any T	Any N	M1	II
**>55 years**	T1	N0/Nx	M0	I
	T1	N1	M0	II
	T2	N0/Nx	M0	I
	T2	N1	M0	II
	T3a/T3b	Any N	M0	II
	T4a	Any N	M0	III
	T4b	Any N	M0	IVA
	any T	any N	M1	IVB

Adapted from [24], with permission from Mary Ann Liebert, Inc., 2020.

**Table 2:** Initial American Thyroid Association (ATA) risk of recurrence classification.

Low Risk	Intermediate Risk	High Risk
All the following are present: -No local or distant metastases -All macroscopic tumor has been resected -No invasion of locoregional tissues -Tumor does not have aggressive histology (e.g., tall cell, insular, columnar cell carcinoma, Hurthle cell carcinoma, follicular thyroid cancer). -No vascular invasion -No ^131^I uptake outside the thyroid bed on the post-treatment scan, if done	**Any of the following is present:** -Microscopic invasion into the perithyroidal soft tissues -Cervical LN metastases or ^131^I uptake outside the thyroid bed on the post-treatment scan done after remnant ablation -Tumor with aggressive histology or vascular invasion	**Any of the following is present:** -Macroscopic tumor invasion -Incomplete tumor resection with gross residual disease - Distant metastases

Reproduced from [25], with permission from Mary Ann Liebert, Inc., 2020.

**Table 3:** Thyroglobulin thresholds for response to therapy and thyroid-stimulating hormone (TSH) targets for each category. RAI, radioactive iodine.

Response to Therapy	Post Total Thyroidectomy with RAI Ablation	Post Total Thyroidectomy without RAI Ablation	Post Hemithyroidectomy	TSH Goals
**Excellent**	Nonstimulated Tg < 0.2 * or stimulated Tg < 1 * and negative imaging	Nonstimulated Tg < 0.2 * or stimulated Tg < 2 * and negative imaging	Nonstimulated Tg < 30 *	0.5–2.0
**Indeterminate**	Nonspecific findings on imaging studies or nonstimulated Tg 0.2–1 or stimulated Tg 1–10, or stable/declining TgAb levels	Nonspecific findings on imaging studies or nonstimulated Tg 0.2–5 or stimulated Tg 2–10, or stable/declining TgAb levels	Nonspecific findings on imaging studies or stable/declining TgAb levels	0.1–0.5
**Structurally Incomplete**	Structural evidence of disease	Structural evidence of disease	Structural evidence of disease	0.1–0.5
**Biochemically Incomplete**	Nonstimulated Tg > 1 or stimulated Tg > 10 or increasing TgAb levels and negative imaging	Nonstimulated Tg > 5 or stimulated Tg > 10 or increasing TgAb levels and negative imaging	Nonstimulated thyroglobulin > 30	<0.1

Tg (ng/mL), TSH (mIU/L); * with undetectable TgAb. Adapted from [34], with permission from Elsevier, 2020.

**Table 4:** The diagnostic criteria for noninvasive follicular thyroid neoplasm with papillary-like nuclear features (NIFTP).

Diagnostic Criteria	Cytomorphology	Histology
Inclusion criteria for diagnosis of NIFTP	-Predominance of microfollicles -Nuclear enlargement, elongation, and overlapping -Nuclear grooves -Nuclear pseudoinclusions -Irregular nuclear contour	-Predominantly follicular pattern -Nuclear features of papillary thyroid carcinoma -Encapsulated or well-demarcated
Exclusion criteria for diagnosis of NIFTP	-Tall cell or columnar cell features -Papillary pattern -Psammoma bodies	->30% solid, insular, or trabecular pattern -Tumor necrosis >3 mitosis/10 high power field -Tall cell or columnar cell features -Papillary pattern -Psammoma bodies -Capsular invasion -Invasion of tumor cells into adjacent uninvolved parenchyma -Lymphatic invasion -Vascular invasion

Reproduced from [39], with permission from Springer Nature, 2020.
